# Reference and Influential Factors of Serum Bone Markers in Chinese Adolescents

**DOI:** 10.1038/s41598-017-17670-x

**Published:** 2017-12-11

**Authors:** Xinyi Wang, Lichao Liu, Ping Li, Jie Ma, Ranhua Jiang, Renee Wang, Ling Li, Haixia Guan

**Affiliations:** 1 Department of Endocrinology and Metabolism, Institute of Endocrinology, Liaoning Provincial Key Laboratory of Endocrine Diseases, The First Hospital of China Medical University, Shenyang, China; 2 Department of Laboratory Medicine, The First Hospital of China Medical University, Shenyang, China; 3 Department of Endocrinology and Metabolism, The Second Hospital of Dalian Medical University, Dalian, China; 4 Department of Endocrinology, Shengjing Hospital of China Medical University, Shenyang, China; 5 Department of Endocrinology, Cangzhou Central Hospital, Changzhou, China; 6 Liaoyang Diabetes Hospital, Liaoyang, China

## Abstract

This study aimed to establish reference ranges of bone markers in Chinese adolescents between the age of 12 and 16, and to search these markers’ characteristics and influential factors. Personal information and fasting blood samples were collected from 174 healthy adolescents in Northeast China. Serum levels of PINP, ALP, β-CrossLaps, calcium, phosphate, PTH, 25(OH)D and TSH were measured. Reference ranges were established for PINP [(85.55–2,028.75)ng/ml], ALP [(53.88–463.63)U/L], β-CrossLaps [(0.16–1.19)ng/ml], calcium [(2.35–2.70)mmol/L], phosphate [(1.17–2.06)mmol/L] and PTH [(2.64–43.36)μg/L] in this population. We observed that bone formation markers PINP and ALP levels were evidently higher when compared to kit references for adults. Reference ranges for calcium, phosphate and PTH also differed from those provided by kit manuals. Serum ALP, PINP, phosphate and PTH changed with age (all P < 0.005), and were significantly higher in boys than in girls (all P < 0.05). Serum 25(OH)D and TSH levels didn’t correlate with PINP, ALP and β-CrossLaps (all P > 0.05). In conclusion, unique reference ranges should be provided for adolescents. BMI, sex and age independently influence certain serum bone markers in adolescents. Vitamin D deficiency is widespread. Serum levels of vitamin D and TSH may not influence bone turnover markers in this population.

## Introduction

Serum bone markers are important indexes to reflect the overall rate of bone formation and resorption. Their measurements can be useful in the assessment of metabolic bone diseases and growth disorder in adolescents. There are a number of common markers used in evaluating bone metabolism. Some of these markers are known as bone formation markers, including bone-specific alkaline phosphatase (BSAP) and procollagen type I N-terminal propertied (PINP). Another group of markers are known as bone resorption markers, such as β-CrossLaps, which is the most sensitive one. Bone mineralization is dependent on adequate intakes of calcium and phosphate, which can be reflected by measuring serum calcium and phosphate levels. Parathyroid hormone (PTH) adjusts the concentration of ionic calcium in blood and is important in bone remodeling. Vitamin D plays a key role in calcium and phosphate metabolism and it is essential for bone health^1^.

In addition, thyroid function are also important in bone maintenance^2^. The hypothalamic–pituitary–thyroid axis plays an key role in skeletal development, acquisition of peak bone mass and regulation of adult bone turnover^3^. Thyroid stimulating hormone (TSH) produced from the axis is a sensitive index of the thyroid function, and has been proposed to be a direct negative regulator of bone turnover in adult^4^. As for our concern, there is limiting research regarding the exact relationship between adolescents’ bone markers and TSH levels.

It should also be noted that bone metabolism in adolescents differs from that of adults. New bones are growing and remodeling faster with growing age, due to high skeletal growth velocity and rapid bone turnover in adolescents. Bone marker references in this specific population may have unique characteristic compared with those of adults^5^, which have not been fully elucidated. These arose our interests in further investigation.

In the present study, we measured bone markers in a group of 12 to 16 years old adolescents in Northeast China, aiming to understand the reference ranges of bone markers such as serum levels of PINP, β-CrossLaps, alkaline phosphatase (ALP, a substitute parameter for BSAP), calcium, phosphate and PTH, in this age group. In addition to age and sex, we examined nutritional status in terms of body mass index (BMI), 25(OH)D and TSH levels for any correlation between these variables and bone markers levels.

## Results

### General data of all subjects

**Figure 1:**
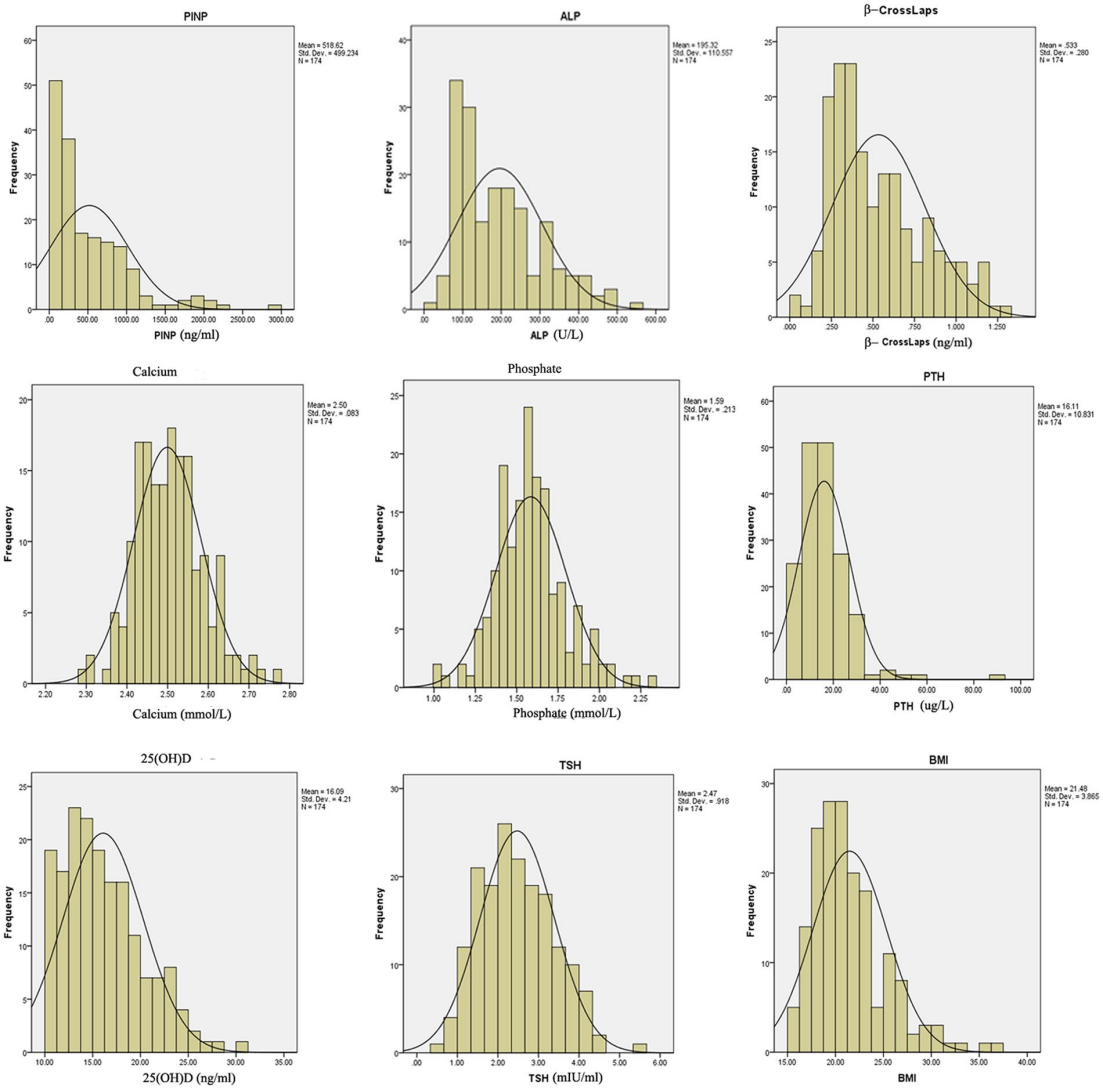
Distribution of parameters measured in 174 adolescents aged from 12 to 16 years.

The general distributions of BMI, bone markers, 25(OH)D and TSH in 174 subjects (82 boys and 92 girls) are shown in Fig. 1. Characteristics of the study population with sex stratification are presented in Table 1. BMI did not show any difference between the two sex groups, despite sex discrepancies in heights and weights.

**Table 1:** Characteristics and bone marker levels in all subjects, stratified by sex.

	**Total**	**Boys**	**Girls**	**P value (boy vs**. **girl)**
Number	174	82	92	—
Height (cm)	162.82 ± 8.15	165.83 ± 9.24	160.14 ± 5.89	0.00*
Weight (kg)	57.25 ± 12.67	59.83 ± 14.50	54.95 ± 10.34	0.01*
BMI (kg/m^2^)	20.76 (18.68, 23.45)	20.84 (18.92, 23.58)	20.75 (18.57, 23.37)	0.73
PINP (ng/ml)	302.40 (153.23, 749.43)	602.10 (408.20, 999.18)	167.10 (126.30, 301.05)	0.00*
ALP (U/L)	176.50 (101.00, 254.00)	220.50 (174.75, 326.25)	108.50 (89.25, 188.50)	0.00*
β-CrossLaps (ng/ml)	0.46 (0.31, 0.69)	0.68 (0.45, 0.94)	0.36 (0.28, 0.49)	0.00*
Calcium (mmol/L)	2.50 ± 0.08	2.49 ± 0.08	2.50 ± 0.09	0.38
Phosphate (mmol/L)	1.59 ± 0.21	1.64 ± 0.23	1.53 ± 0.18	0.00*
PTH (μg/L)	14.97 (8.93, 21. 20)	17.17 (9.87, 25.50)	13.35 (8.20, 16.46)	0.00*
25(OH)D (ng/ml)	16.09 ± 4.21	16.33 ± 4.70	15.88 ± 3.74	0.48
TSH (mIU/ml)	2.47 ± 0.92	2.44 ± 0.98	2.50 ± 0.87	0.65

BMI: body mass index; PINP: procollagen 1 N-terminal peptide; ALP: alkaline phosphatase; PTH: parathyroid hormone; 25(OH)D: 25-hydroxy vitamin D; TSH: thyroid stimulating hormone. Parametric data [height, weight, calcium, phosphate, 25(OH)D and TSH] are reported as mean ± SD. Nonparametric data (BMI, PINP, ALP, β-CrossLaps and PTH) are reported as medians with the range between 25^th^ and 75^th^ percentiles. *Statistics significance between boys and girls, *P < 0.05.

### Reference ranges for bone markers in the adolescent population and comparison with commercial kit references

Reference ranges of bone markers in this adolescent population were defined as the central 95%, between 2.5^th^ and 97.5^th^ percentiles of serum concentrations. Shown in Table 2, we observed that bone formation markers PINP (85.55ng/ml-2,028.75ng/ml) and ALP (53.88U/L-463.63U/L) levels were evidently higher when compared to kit references for adults (15.13ng/ml-76.31ng/ml and 35.00U/L-130.00U/L, respectively). Reference ranges for calcium, phosphate and PTH also differed from ranges provided by kit manuals (Table 2).

**Table 2:** Reference ranges for bone markers in adolescents and comparison with commercial kit references.

2.5^th^−97.5^th^ Percentiles*	PINP (ng/ml)	ALP (U/L)	β-CrossLaps (ng/ml)	Calcium (mmol/L)	Phosphate (mmol/L)	PTH (μg/L)
	85.55–2,028.75	53.88–463.63	0.16–1.19	2.35–2.70	1.17–2.06	2.64–43.36
Kit References						
Manual version	2013–10, V11.0	2015–02, V5.0	2013–10, V13.0	2014–02, V3.0	2015–07, V7.0	2013–11, V23.0
Male	n/a	40–130	(30–50 years) < 0.584	(18–60 years) 2.15–2.50	0.81–1.45	15–65
Female	(30–89 years) Post-menopause 20.25–76.31 Pre-menopause 15.13–58.59	35–105	(30–89 years) Post-menopause < 1.008 Pre-menopause < 0.573	(18–60 years) 2.15–2.50	0.81–1.45	15–65
Adolescent	n/a	Male (13–17 years) < 390 Female (13–17 years) < 187	n/a	(12–18 years) 2.10–2.55	Male (13–15 years) 0.95–1.65 Female (13–15 years) 0.90–1.55	n/a

^*^Reference intervals obtained in our study are presented as the central 95%, between2.5^th^ and 97.5^th^ percentiles of serum concentrations. PINP: procollagen 1 N-terminal peptide; ALP: alkaline phosphatase; PTH: parathyroid hormone; n/a: not available.

### Bone markers stratified by sex and age

From our results (Table 1), bone markers observed varied with sex difference. PINP, β-CrossLaps, ALP, phosphate and PTH levels were significantly higher in the group of boys (all P < 0.05), while the remainder indexes that were examined did not show apparent differences.

**Figure 2:**
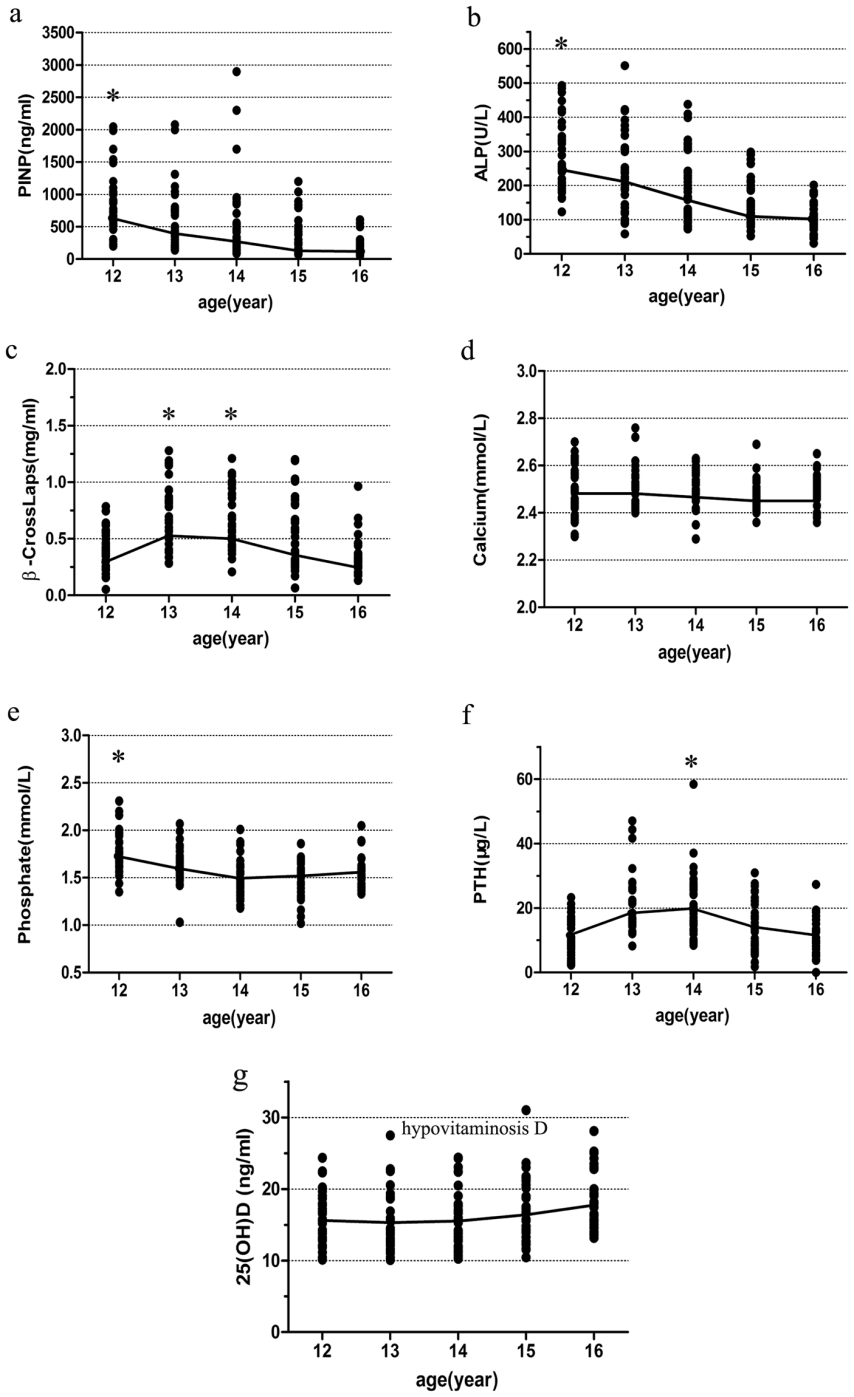
Scatterplot of the bone markers values (**a–g**), based on age stratification from 12 to 16 years old. (**a**) *PINP level in 12-year-old group is the highest when compared with 14, 15 and 16-year-old groups (P < 0.005). No statistical significance between 12 and 13-year-old groups. (**b**) *ALP level in 12-year-old group is the highest when compared with 14, 15 and 16-year-old groups (P < 0.005). No statistical significance between 12 and 13-year-old groups. (**c**) *β-CrossLaps levels in 13 and 14 year-old groups are significantly higher than those in 12 and 16 year-old groups (P < 0.005). (**e**) *Serum phosphate levels are significantly lower in adolescents over 12 years of age compared to adolescents of 12 years old (P < 0.005). (**f**) *Serum PTH level in 14-year-old group is the highest when compared with 12, 15 and 16 age groups (P < 0.005). No statistical significance between 13 and 14-year-old groups. (**g**) 25(OH)D < 30 ng/ml defined as hypovitaminosis D.

Clinical characteristics of the adolescents, with age stratifications, are listed in Table 3. All bone markers variation tendencies are shown in Fig. 2. Our results indicated that serum PINP and ALP levels decreased significantly with increased age, measured from [781.50 (542.50, 1,035.50)]ng/ml and [263.00 (214.00, 343.50)]U/L in 12-year-old adolescents, to [144.75 (112.83, 216.03)]ng/ml and [104.00 (84.75, 146.00)]U/L in 16-year-old adolescents, respectively. Neither PINP nor ALP levels showed significant differences between 12-year-old and 13-year-old adolescent groups, while results in these two groups were higher than those in 14, 15 and 16 year-old groups (all P < 0.005). Serum β-CrossLaps levels presented an inverted U-shaped association with age. Results of measurements in 13 and 14 year-old groups were significantly higher than those in 12 and 16 year-old groups (all P < 0.005). Serum phosphate levels were notably lower in groups above the age of 12 when compared to levels observed in 12-year-old adolescents (1.77 mmol/L ± 0.21 mmol/L) (all P < 0.005). Serum PTH levels were the highest in 14-year-old adolescents [20.04 (15.02, 26.51)] μg/L when compared with 12, 15 and 16 age groups (all P < 0.005). Serum calcium levels and serum 25(OH)D levels were not significantly different across all age groups (all P > 0.005). It should be mentioned that majority of the 25(OH)D levels measured across all age groups were well below recommended 25(OH)D level provided by the Endocrine Society^6^ (Fig. 2g), although severe vitamin D deficiency with 25(OH)D < 10 ng/ml had already been taken out of consideration.

**Table 3:** Characteristics and bone marker levels in all subjects, stratified by age.

Age	12yr	13yr	14yr	15yr	16yr	P value
N (boy:girl)	37(18:19)	33 (15:18)	38(19:19)	36(18:18)	30(12:18)	–
Height (cm)	155.58 ± 6.76	163.67 ± 8.49	164.24 ± 6.78	166.07 ± 6.57	165.13 ± 7.73	0.00*
Weight (kg)	49.97 ± 11.90	57.67 ± 10.48	59.18 ± 10.78	60.94 ± 14.91	58.90 ± 12.32	0.00*
BMI (kg/m^2^)	19.63 (17.76, 22.58)	21.30 (18.52, 23.82)	21.30 (19.50, 23.63)	20.88 (19.00, 23.53)	20.40 (19.20, 23.45)	0.51
PINP (ng/ml)	781.50 (542.50, 1,035.50)	487.60 (238.25, 795.65)	332.55 (164.63, 598.00)	157.40 (111.65, 410.83)	144.75 (112.83, 216.03)	0.00*
ALP (U/L)	263.00 (214.00, 343.50)	225.00 (129.50, 309.00)	166.00 (95.75, 239.25)	113.50 (89.25, 190.50)	104.00 (84.75, 146.00)	0.00*
β-CrossLaps (ng/ml)	0.37 (0.24, 0.54)	0.66 (0.43, 0.86)	0.63 (0.49, 0.91)	0.45 (0.28, 0.77)	0.31 (0.26, 0.37)	0.00*
Calcium (mmol/L)	2.52 ± 0.11	2.52 ± 0.09	2.50 ± 0.07	2.48 ± 0.07	2.48 ± 0.07	0.16
Phosphate (mmol/L)	1.77 ± 0.21	1.61 ± 0.19	1.48 ± 0.19	1.51 ± 0.18	1.56 ± 0.16	0.00*
PTH (μg/L)	9.72 (6.77, 15.11)	18.30 (15.15, 26.20)	20.04 (15.02, 26.51)	12.94 (6.82, 20.50)	9.78 (6.03, 13.89)	0.00*
25(OH)D (ng/ml)	15.50 ± 3.61	15.15 ± 4.38	15.41 ± 4.11	16.51 ± 4.35	18.21 ± 4.12	0.02*
TSH (mIU/ml)	2.80 ± 0.98	2.26 ± 0.77	2.51 ± 0.84	2.22 ± 0.95	2.57 ± 0.97	0.05

BMI: body mass index; PINP: procollagen 1 N-terminal peptide; ALP: alkaline phosphatase; PTH: parathyroid hormone; 25(OH)D: 25-hydroxy vitamin D; TSH: thyroid stimulating hormone. Parametric data (height, weight, calcium, phosphate, 25(OH)D and TSH) are reported as mean ± SD. Nonparametric data (BMI, PINP, ALP, β-CrossLaps and PTH) are reported as medians with the range between 25^th^ and 75^th^ percentiles. The parametric variables were compared using one-way ANOVA test and logarithmic or square root transformations were applied as needed to achieve a distribution as close as possible to normal. *P < 0.05.

### Influential factors of serum bone markers

In addition to sex and age, serum 25(OH)D and TSH were analyzed in correlation with bone markers in adolescents. Results are shown in Table 4. Serum 25(OH)D level negatively correlated with PTH levels, but the correlation was weak (r = − 0.20, P = 0.01). There were no significant associations between 25(OH)D and serum PINP, ALP, β-CrossLaps, calcium or phosphate levels. There were no associations between serum TSH level and any bone marker levels (all P > 0.05) either.

**Table 4:** Correlations of bone markers with serum 25(OH)D and TSH.

	25(OH)D		TSH	
	Correlation coefficient (r)	Significance (P)	Correlation coefficient (r)	Significance (P)
Calcium (mmol/L)	0.09	0.24	0.10	0.19
Phosphate (mmol/L)	0.00	0.97	0.14	0.06
PTH (μg/L)	−0.20	0.01*	−0.02	0.85
PINP (ng/ml)	−0.09	0.26	0.00	0.96
ALP (U/L)	−0.11	0.16	−0.02	0.85
β-CrossLaps (ng/ml)	−0.05	0.51	−0.09	0.24

25(OH)D: 25-hydroxy vitamin D; TSH: thyroid stimulating hormone; PTH: parathyroid hormone; PINP: procollagen 1 N-terminal peptide; ALP: alkaline phosphatase. Pearson and Spearman correlation coefficients were used to find correlations between parametric and nonparametric data, respectively. Parametric data include calcium, phosphate, 25(OH)D and TSH; nonparametric data include PINP, ALP, β-CrossLaps and PTH. *P < 0.05.

Multiple regression analysis was used to identify covariates that might influence bone turnover marker values. The covariates included BMI, age, sex, 25(OH)D and TSH. Results are shown in Table 5. BMI was an independent predictor of serum PINP level (P = 0.01). Age was an independent predictor of ALP and PINP levels (both P = 0.00). Sex was an independent predictor of ALP, PINP and β-CrossLaps levels (all P = 0.00). TSH and 25(OH)D were not influential factors of bone markers in this adolescent population.

**Table 5:** Multiple regression analysis of factors influencing serum bone remodeling markers.

Bone markers		Coefficients beta	Standard error	Significance
PINP	Age	−0.44	20.97	0.00*
	Sex	−0.46	56.75	0.00*
	BMI	−0.16	7.38	0.01*
	25(OH)D	−0.03	6.9	0.56
	TSH	−0.05	31.07	0.36
β-CrossLaps	Age	−0.07	0.01	0.30
	Sex	−0.54	0.04	0.00*
	BMI	0.06	0.01	0.37
	25(OH)D	−0.19	0.00	0.78
	TSH	−0.07	0.02	0.29
ALP	Age	−0.56	4.23	0.00*
	Sex	−0.47	11.44	0.00*
	BMI	−0.04	1.49	0.42
	25(OH)D	0.00	1.39	0.96
	TSH	−0.04	6.27	0.46

PINP: procollagen 1 N-terminal peptide; ALP: alkaline phosphatase; BMI: body mass index; 25(OH)D: 25-hydroxy vitamin D; TSH: thyroid stimulating hormone;

*P < 0.05.

## Discussion

In this study, we established serum bone marker references within healthy Chinese adolescents between the aged of 12 to 16. These markers included bone formation markers (PINP), bone resorption marker (β-CrossLaps), ALP, bone related mineralization markers (calcium and phosphate) and PTH. In fact, compared with BSAP, ALP is not a sensitive and specific parameter to reflect bone metabolism, because ALP includes not only BSAP but also liver alkaline phosphatase. Nonetheless, when liver alkaline phosphatase is stable and remains within normal value, ALP presents the same trend as BSAP. Given the fact that ALP measurement is easier to obtain, cost-effective and more commonly used in China, it was examined and analyzed in the present study. For PINP, β-CrossLaps and PTH, kit manuals do not provide specific reference ranges for these markers in adolescents, neither do they mention the applicability of kit reference ranges to adolescent population. For ALP, calcium and phosphate, the kit manuals recommend reference ranges for both adults and adolescents, however, these ranges were not evaluated by the kit for they were quoted from other publications. We found that in our adolescent population, reference ranges of serum PINP and ALP were significantly higher than the kit’s reference for adults, and reference ranges of calcium, phosphate and PTH also differed from those provided by the kit’s manufacturer. As a result, reference ranges of most bone markers proposed in kit manuals are not suitable for evaluating adolescents. The present study suggests the necessity of establishing unique reference intervals for healthy adolescents in laboratories.

Of all the factors that may influence bone markers in adolescents, we found that sex difference played a role in bone marker concentrations. Serum ALP, PINP, β-CrossLaps, phosphate and PTH levels in boys were higher than the levels in girls, suggesting a strong relation of these markers to the later male pubertal growth spurt, as has been shown by others^7^. According to previous publications, growth spurt occurs approximately between the age from 10 to 12 in girls and 12 to 14 in boys^5,8^. After the period of accelerated growth, biochemical markers of bone remodeling decrease but boys marker remain higher than in age-matched girls^9^.

This study also showed that serum levels of the bone markers ALP and PINP were the highest in age 12–13 and significantly decreased with increased age in adolescents. This results are in accordance with previous reports^10^. Age is another important variable that affects concentrations of bone formation markers in adolescents^11^. There are two peaks of bone growth during childhood development. The first peak appears during infancy; the second peak occurs during early puberty. Recent study found that bone markers increased rapidly during early puberty when growth velocity was highest and the period of most rapid bone mineral accrual^12,13^. Being indexes in the association with rapid growth velocities in healthy people, decreases in serum ALP and PINP as individuals growing older after early puberty come as no surprise.

Serum 25(OH)D is an index evaluating vitamin D nutrition status. According to the Endocrine Society’s guideline, vitamin D deficiency is defined as 25(OH)D below 20 ng/ml (50 nmol/L), vitamin D insufficiency is defined as 25(OH)D between 21 and 29 ng/ml (52.5–72.5 nmol/L), and vitamin D sufficiency is defined as 25(OH)D above 30 ng/ml (75 nmol/L). Thus, hypovitaminosis D is defined as 25(OH)D below 30 ng/ml (75 nmol/L)^14^. Based on this criteria, the phenomenon of widespread vitamin D deficiency among Northeast Chinese adolescents has been observed in our study, with majority of recorded 25(OH)D levels being under 20 ng/ml (50 nmol/L). This result can not represent the entire Chinese adolescent population, because we only recorded 25(OH)D levels from adolescents aged from 12 to 16 in Northeast China, but similar findings have been demonstrated in another study which concluded that Chinese children tend to be vitamin D deficient^15^. As previously reported, lack of outdoor activities and inadequate sunshine exposure are main reasons caused most low level of 25(OH)D^16^. Usages of sunscreen, the short sunshine duration in the north of China and lack of purchasing channels of vitamin supplements are also notable predictors of vitamin D deficiency^15,17^. These may explain the results obtained from the present study. The phenomenon of widespread vitamin D deficiency among adolescents worldwide^18^ makes it difficult to find a perfectly normal population, in which sufficient nutrition status is achieved, for building up reference ranges for bone markers. However, it should be noted that controversies remain in how to define the “reference range” of vitamin D, and remain in whether the criteria of hypovitaminosis D proposed by the Endocrine Society is applicable to all population^19^. In the present study, following the method used in previous study^20^, we did not include results from subjects with 25(OH)D < 10 ng/ml in final analysis, in order to avoid marked impacts that may be caused by severe vitamin D deficiency. Excluding these subjects did not significantly change the results.

Our analysis suggested that serum 25(OH)D level was weakly negatively correlated to PTH level, but had no correlation with calcium and phosphate. This finding is in accordance with the well-adapted knowledge that vitamin D is not the only regulatory factor for calcium and phosphorus metabolism. Vitamin D co-acts with PTH hormone to be involved in the complex bone-kidney-parathyroid loop^21^. By promoting calcium and phosphate absorption in intestines, as well as increasing osteoclast numbers and stimulating bone resorption, vitamin D and PTH work together to maintain serum calcium and phosphate relatively stable, which is very important for bone development in adolescents^22^. However, it should be noted that serum 25(OH)D measurements are not suitable for bone turnover follow-up in this particular adolescent population, not only because of the high prevalence of vitamin D insufficient, but also due to lack of correlation between 25(OH)D and bone turnover markers.

Finally, our study showed no correlation between serum TSH levels and bone markers in healthy adolescents. Previous studies only demonstrated that low-normal TSH values were associated with an increased prevalence of vertebral fractures in post-menopausal women^23^, few studies proved the effects of thyroid hormones and TSH on bones in terms of bone markers. Normal level of thyroid hormone is important in keeping bone health, even subclinical hyperthyroidism, defined by a suppressed TSH level in the presence of normal thyroid hormone concentrations, is associated with fracture^24^. TSH receptor (TSHR) has been demonstrated in both osteoblasts and osteoclasts, suggesting TSH may exert direct action in these cells^4^. Thus, we suspected thyroid function may affect bone markers in adolescents. However, in our study, we did not find the correlation we expected. Further researches on this topic will benefit from a larger sample size.

Some limitations do exist, despite the fact that present study has provided relatively thorough information on bone markers in adolescents. First of all, BSAP, instead of total ALP, should be detected as a more sensitive bone formation marker. Secondly, we did not obtain data on Tanner staging, which helps to evaluate puberty in adolescents, thus we could not accurately assess the association between early puberty and bone markers in the studied population. Thirdly, in view of our current results, to establish accurate age and sex-specific reference ranges in adolescents should be considered. Unfortunately, our study could not reach this goal, because subject number of each age and sex-specific group was not big enough to set up a reference range. Lastly, we did not collect information on outdoor activities and sunshine exposure of these adolescents, so we could not elucidate real reasons for their vitamin D deficiency. These limitations need to be improved in future in-depth studies.

In conclusion, we have established reference ranges for bone markers PINP [(85.55–2,028.75)ng/ml], ALP [(53.88–463.63)U/L], β-CrossLaps [(0.16–1.19)ng/ml], calcium [(2.35–2.70)mmol/L], phosphate [(1.17–2.06) mmol/L] and PTH [(2.64–43.36)μg/L] in an adolescent population from Northeast China. References provided by kits can not apply to this specific population. BMI, sex and age independently influence certain serum bone markers in adolescents. Vitamin D deficiency is widespread. Serum levels of vitamin D showed weakly negative correlation to PTH, but along with serum TSH, may not affect bone turnover markers in this population.

## Methods

### Subjects

One thousand, three hundred and twelve students from middle schools were recruited for the previous epidemiological investigation. The investigation took place in Anshan, Northeast China. Using stratified cluster-sampling method, we initially selected 196 adolescents from them for the present study. The target population was aged from 12 to 16 years old^25^. Demographic information and medical history, including sex, age, previous medications intake, as well as vitamins and other supplements intakes were collected from each subject. The final analysis excluded 22 subjects, including one who suffered a bone fracture within 6 months; two who took vitamin D and calcium supplements within 3 months; two with history of autoimmune thyroid diseases; one with type 1 diabetes; and sixteen with serum 25(OH)D < 10ng/ml, due to the concern that severe vitamin D deficiency would affect reference ranges of bone markers^20^. Results from a total of 174 adolescents (82 boys and 92 girls) were eventually reported. Height and weight were measured during recruitment between December 2010 and March 2011, and body mass index (BMI) was calculated as an indicator for nutritional status.

### Blood samples collection

Fasting blood samples (5–10 ml) were collected from each subject between 7:00am and 9:00am to minimize the effects of the diurnal variation of bone remodeling markers. Serum separations were completed within 2 hours upon collection (3000r/10 min). Aliquots of serum were stored at −80 °C until tests were run. Sample collections and separations were performed according to standard laboratory procedures.

### Laboratory assays

Serum PINP, β-CrossLaps, 25(OH)D, PTH and TSH levels were determined by electrochemiluminescence immunoassays (ECLA) using Roche commercial kits (COBAS e 601, Roche, Germany). Serum ALP, calcium and phosphate levels were determined using the Roche P-modular automated analyzer (Roche, Germany). All measures were done within two days in June, 2014. Intra- and inter-assay coefficients of variation of all the commercial kits were < 10%.

### Statistical analysis

Data were analyzed using SPSS software (SPSS 20.0, IBM, USA). Parametric data were reported as mean ± SD. Nonparametric data were reported as median with interquartile range. Bone marker concentrations were tested for their normal distribution. Logarithmic or square root transformations were applied to variables as needed to achieve a distribution as close as possible to normal. The parametric variables were compared using t-test between two groups and Kruskal-Wallis one-way ANOVA test among multiple groups, respectively. Pearson and Spearman correlation coefficients were used to find correlation between parametric and nonparametric variables, respectively. P level of 0.005 was considered significant for multiple comparisons among 5 groups when necessary^26^. P value of 0.05 was used otherwise. Reference ranges of serum bone markers were defined as the central 95%, ranged between the 2.5^th^ and 97.5^th^ percentiles of their concentrations^20^.

### Ethics

Ethical approval was obtained from the Research Ethics Committee of Shengjing Hospital of China Medical University and methods were conducted in accordance with approved guidelines and regulations. All subjects and subject’s parents or guardians from the cohort provided his or her written informed consents for using their data and blood samples.

### Data availability

The datasets generated during and/or analysed during the current study are available from the corresponding author on reasonable request.
